# Reducing inequities in maternal and child health in rural Guatemala through the CBIO+ approach of Curamericas: 2. Study site, design, and methods

**DOI:** 10.1186/s12939-022-01754-w

**Published:** 2023-02-28

**Authors:** Henry B. Perry, Mario Valdez, Stanley Blanco, Ramiro Llanque, Shayanne Martin, Jason Lambden, Corey Gregg, Kaitlin Leach, Elijah Olivas, Barbara Muffoletto, Jacqueline Wallace, Nina Modanlo, Erin Pfeiffer, Carey C. Westgate, Breanne Lesnar, Ira Stollak

**Affiliations:** 1 Health Systems Program, Department of International Health, Johns Hopkins School of Public Health, Baltimore, Maryland USA; 2 Curamericas/Guatemala, Calhuitz, San Sebastián Coatán, Huehuetenango, Guatemala; 3 Consejo de Salud Rural Andino/Curamericas, La Paz, Bolivia; 4 Institute for Global Health Sciences, University of California San Francisco, San Francisco, California USA; 5 McGaw Medical Center, Northwestern University, Chicago, Illinois USA; 6 Department of Internal Medicine, Louisiana State University Health Sciences Center at New Orleans (LSUHSC-NO), New Orleans, Louisiana USA; 7 Optum, SeaTac, Washington USA; 8 Student, PhD Program, Department of International Health, Johns Hopkins School of Public Health, Baltimore, Maryland USA; 9 Curamericas Global, Raleigh, North Carolina USA; 10 Independent Consultant, Baltimore, Maryland USA; 11 David Geffen School of Medicine, University of California Los Angeles, Los Angeles, California USA; 12 Independent Consultant, Winston-Salem, North Carolina USA; 13 Community Health Impact Coalition, New York, New York USA; 14 Program Coordinator for Research Engagement, AVAC (Global Advocacy for HIV Prevention), New York City, New York USA

**Keywords:** Implementation research, Maternal health, Child health, Community health, Primary health care, Community-based primary health care, Census-based, Impact-oriented approach, Care Groups, Community Birthing Centers, Guatemala, Equity, Curamericas Global, Curamericas/Guatemala

## Abstract

**Background:**

The Curamericas/Guatemala Maternal and Child Health Project, 2011–2015, included implementation research designed to assess the effectiveness of an approach referred to as CBIO+﻿ , composed of: (1) the Census-Based, Impact-Oriented (CBIO) Approach, (2) the Care Group Approach, and (3) the Community Birthing Center Approach*.* This is the second paper in a supplement of 10 articles describing the implementation research and its findings. Paper 1 describes CBIO+ , the Project Area, and how the Project was implemented.

**Objective:**

This paper describes the implementation research design and details of how it was carried out.

**Methods:**

We reviewed the original implementation research protocol and the methods used for all data collection related to this Project. The protocol and methods used for the implementation research related to this Project were all standard approaches to the monitoring and evaluation of child survival projects as developed by the United States Agency for International Development Child Survival and Health Grants Program (CSHGP) and the CORE Group. They underwent independent peer review supervised by the CSHGP before the implementation research began.

**Results:**

The study area was divided into two sets of communities with a total population of 98,000 people. Project interventions were implemented in Area A from 2011 until the end of the project in 2015 (44 months) and in Area B from late 2013 until 2015 (20 months). Thus, Area B served as a quasi-comparison area during the first two years of Project implementation. The overarching study question was whether the CBIO+ Approach improved the health and well-being of children and mothers. The outcome indicators included (1) changes in population coverage of evidence-based interventions, (2) changes in childhood nutritional status, (3) changes in the mortality of children and mothers, (4) quality of care provided at Community Birthing Centers, (5) the impact of the Project on women’s empowerment and social capital, (6) stakeholder assessment of the effectiveness of the CBIO+ Approach, and (7) the potential of wider adoption of the CBIO+ Approach.

**Conclusion:**

The implementation research protocol guided the assessment of the effectiveness of the CBIO+ Approach in improving the health and well-being of children, mothers, and their communities.

## Background

From 2011 to 2015, Curamericas/Guatemala implemented the Maternal and Child Health Project (hereafter referred to as the Project) that included implementation research designed to assess the effectiveness of a combined approach composed of: (1) the Census-Based, Impact-Oriented (CBIO) Approach, (2) the Care Group Approach, and (3) the Community Birthing Center Approach. This is the second paper in a supplement of 10 articles describing the implementation research and its findings. The supplement provides a more readily accessible analysis and reporting of the findings of the implementation research that were carried out for this Project and that were submitted in its complete form to the United States Agency for International Development at the completion of the Project [1].

The first paper in the supplement describes the approaches used by the Project in the Cuchumatanes mountains of the Western Highlands of Guatemala, in the Department of Huehuetenango, along with the physical and social context in which the Project was implemented [2]. In this second paper, we describe the study sites for the implementation research, the implementation research design, and the methods used. The subsequent eight papers in this supplement [3–10] describe the findings and implications of the implementation research.

### Study sites

**Fig. 1:**
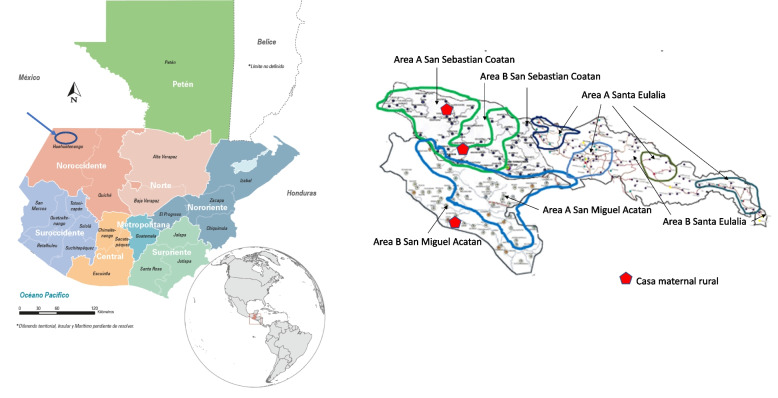
Maps showing the location of the Project Area in Guatemala (left) and Areas A and B of implementation in their respective municipalities along with the location of the three Community Birthing Centers (*Casas Maternas Rurales*) that were operating during the time of Project implementation (right)

The difficult and extensive mountainous terrain, together with limited financial and human resources, as described in Paper 1, required that the Project be implemented in two phases. During the first two years (October 2011 to September 2013), the Project implemented interventions in 89 communities. These first two years were designated as Phase 1 and these 89 communities constitute Area A. In the final 20 months (October 2013 to May 2015), designated as Phase 2, the Project expanded to an additional 91 communities that constitute Area B. Because the communities of Area A were generally further from existing *Ministerio de Salud Pública y Asistencia Social (*MSPAS, or Ministry of Public Health and Social Assistance) clinics, they were prioritized for Project services and continued to receive services for the full 44-month duration of the Project. Areas A and B were adjacent as well as geographically and socio-culturally similar. Areas A and B each included approximately half of the geographic area and half of the population of the three municipalities comprising the Project Area. Figure 1 below contains a map of the three municipalities delineating the boundaries of the two Phases in each municipality.

## Methods

We reviewed all documents developed for this Project, including the original Project proposal, the accompanying implementation research proposal, the Detailed Implementation Plan, and the monitoring and evaluation (M&E) reports. Most of this information is contained in the Project’s final evaluation, available elsewhere [1]. After reviewing these documents, we summarized the research questions, indicators to assess impact, quantitative and qualitative data collection procedures, and ethical approval. This summary is described below. The protocol and methods used for the implementation research related to this Project were all standard approaches to the monitoring and evaluation of child survival projects as developed by the United States Agency for International Development Child Survival and Health Grants Program [11] and the CORE Group [12] and underwent independent peer review by a panel of experts convened by the Program before the implementation research began.

## Results

### Rationale for the research questions

The implementation research built on past studies of the CBIO Approach, the Care Group Approach, and the Community Birthing Center Approach (which involved the development and implementation of *Casas Maternas Rurales,* hereafter referred to as Birthing Centers). The learning objective was to assess the potential synergy of the CBIO+ Approach within the existing Guatemalan rural health system. This was done by measuring the health and social impacts of the combined approach which was implemented together with the Guatemalan public-sector initiative known as the Program for Extension of Coverage (*Programa de Extensión de Cobertura,* or PEC), described in Paper 1 [2]. Health impact was measured by changes in health behaviors, childhood nutritional status, and mortality while social impact was measured by changes in the empowerment of women and social capital of communities in the Project Area. Cost-effectiveness of the CBIO+ Approach was also assessed by measuring implementation costs, cost per life saved, and cost per disability-adjusted-life years (DALYs) averted.

Past anecdotal experience suggested that the CBIO Approach empowered program staff and participating communities while the Care Group approach appeared to empower not only the women who served as Care Group Volunteers but also their beneficiaries as well (Melanie Morrow and Thomas Davis, personal communication, 2012). Consequently, well-being, as a combination of women’s empowerment and social capital, was included as an indicator in this study.

### Hypotheses and research questions

The implementation research was designed to test the following hypotheses:

1. The CBIO+ Approach improves the population coverage of interventions that are designed to address the epidemiological priorities for mothers and children relative to (a) baseline measures of these indicators, (b) measures in a comparison area (Area B), (c) measures in selected nearby municipalities where the Project was not implemented, and (d) the overall rural population of the Department of Huehuetenango. Paper 3 [3] in this supplement addresses this hypothesis.
2. The CBIO+ Approach improves the nutritional status of children relative to (a) baseline measures of these indicators, (b) measures in a comparison area (Area B), (c) measures in selected nearby municipalities where the Project was not implemented, and (d) the overall rural population of the Department of Huehuetenango. Paper 4 [4] in this supplement addresses this hypothesis.
3. The CBIO+ Approach reduces mortality of children younger than 5 years of age (hereafter referred to as under-5 mortality) and maternal mortality relative to (a) baseline measures of these indicators, (b) measures in a comparison area (Area B), (c) measures in selected nearby municipalities where the Project was not implemented, and (d) the overall rural population of the Department of Huehuetenango. Paper 5 [5] in this supplement addresses this hypothesis.
4. The Community Birthing Center Approach provides mothers with a safer alternative to home delivery that is also culturally appropriate in the local context. Paper 6 [6] addresses this hypothesis.
5. The CBIO+ Approach empowers women engaged as volunteers and as beneficiaries, and it improves self-esteem and decision-making autonomy. It also builds social capital. Papers 7 [7] and 8 [8] address this hypothesis.
6. Stakeholders, including Project beneficiaries, community leaders, Project staff and MSPAS staff, consider the CBIO+ Approach to be an effective and appropriate improvement to programs for improving the health and well-being of children and their mothers. Paper 9 [9] addresses this hypothesis.
7. The CBIO+ Approach is affordable and cost-effective for Guatemala, thus meriting consideration for scale up in other parts of Guatemala as well as for implementation and testing in areas of the world where resources are highly constrained, access to healthcare is difficult, health services are limited, and the burden of disease among children and mothers remains high. Paper 10 [10] addresses this hypothesis.

### Indicators

Table 1 summarizes the Project’s monitoring and evaluation (M&E) system. The table describes, for each type of data, the source, how often it was collected, by whom it was collected, and where the data were recorded.

**Table 1:** The Project monitoring and evaluation system: topic, indicators, data sources, data collectors, frequency of data collection, and data record

Topic	Indicators	Data source(s)	Data collector(s)	Date of collection/frequency	Location of data
Outputs of Project activities	Number of mothers of under-2 children reached by Care Group Approach Number of Community Health Committees established and trained Number of Level-1 and Level-2 Promoters, Care Group Volunteers and *comadronas* trained Number of Care Groups and Self-Help Groups formed Number of women participating in Self-Help Groups Number of children participating in Positive Deviance/Hearth workshops Number of women delivering in health facilities Number of household visitations for child growth monitoring and vitamin A supplementation Number of children treated for diarrhea and pneumonia Number of children vaccinated Number of children supplemented with vitamin A Number of children receiving growth monitoring	Logs (*actas)* of Project activities Attendance records of Care Groups, Self-Help Groups, *Círculos*, and Positive Deviance/Hearth workshops Community Registers Logs of home visitations	Level-1 and Level-2 Promoters	Monthly (ongoing)	Microsoft Excel database of Project M&E system
Child nutritional status	Weight-for-age Z scores Height-for-age Z scores Weight-for-height Z scores	KPC surveys Anthropometric censuses (*“barridos*”) Regular child growth monitoring visits to households	Contracted interviewers supervised by Project staff (Jan 2012 and June 2015) Level-2 Promoters (Sept 2012) Level-1 and Level-2 Promoters	Jan 2012 (both Areas) Sept 2012 (Area A only) June 2015 (both Areas) Every 3 to 6 months 2012–2014 Monthly (ongoing)	EPI INFO and Excel data bases
Vital events (newly identified pregnancies, births, stillbirths, and deaths)	Neonatal mortality rate 1–11-month mortality rate 12–59-month mortality rate Maternal mortality ratio Stillbirth rate	Self-Help Group and Care Group meetings; routine home visits	Care Group Volunteers, Level-1 and Level-2 Promoters, Care Group Supervisors	Monthly (ongoing)	Vital Events Registers and Community Registers
Causes of mortality and contributing factors	Causes of death Barriers to seeking and obtaining medical care Place of death Place of delivery (for maternal and neonatal deaths) Timing of neonatal deaths (1^st^ 24 h, 1^st^ week, 2^nd^-4^th^ weeks)	Verbal autopsies	Institutional Facilitators	Monthly (ongoing)	Verbal autopsy reports and Vital Events Registers
Coverage of antenatal care interventions	Antenatal care utilization Immunization coverage (tetanus) Iron/folate consumption during pregnancy Knowledge of at least 2 danger signs during pregnancy	KPC surveys	Contracted interviewers supervised by Project staff	Jan 2012 and June 2015	EPI INFO and Excel data bases
Coverage of delivery care interventions	Knowledge of at least 2 danger signs during labor/delivery Presence of an emergency transport system for pregnancy and/or newborn complications Delivery by a skilled health worker Delivery at a health facility	KPC surveys	Contracted interviewers supervised by Project staff	Jan 2012 and June 2015	EPI INFO and Excel data bases
Coverage of postpartum care interventions	Knowledge of at least 2 postpartum danger signs Postpartum visit within 48 h after birth	KPC surveys	Contracted interviewers supervised by Project staff	Jan 2012 and June 2015	EPI INFO and Excel data bases
Coverage of newborn care interventions	Knowledge of at least 2 danger signs in neonates Essential newborn care provided (cleaning of umbilical cord, immediate breastfeeding, and thermal care)	KPC surveys	Contracted interviewers supervised by Project staff	Jan 2012 and June 2015	EPI INFO and Excel data bases
Coverage of birth spacing and family planning interventions	Knowledge of at least 2 risks associated with a pregnancy interval of < 24 months Current use of modern contraceptives among non-pregnant women Birth interval > 24 months between two previous deliveries	KPC surveys	Contracted interviewers supervised by Project staff	Jan 2012 and June 2015	EPI INFO and Excel data bases
Coverage of child feeding interventions	Exclusive breastfeeding (children 0- < 6 months of age) in previous 24 h Proper infant and young child feeding (children 6- < 24 months of age) Vitamin A supplementation (children 2- < 24 months of age) in previous 6 months	KPC surveys	Contracted interviewers supervised by Project staff	Jan 2012 and June 2015	EPI INFO and Excel data bases
Coverage of childhood pneumonia interventions	Children with cough and rapid/difficult breathing in the previous 2 weeks Appropriate care-seeking for child with symptoms of pneumonia	KPC surveys	Contracted interviewers supervised by Project staff	Jan 2012 and June 2015	EPI INFO and Excel data bases
Coverage of childhood diarrhea interventions	Children with diarrhea episode in the previous 2 weeks Use of oral rehydration solution (including a recommended home fluid) during a diarrheal episode Use of zinc during a diarrheal episode Increased fluid intake during a diarrheal episode Increase food intake following a diarrheal episode	KPC surveys	Contracted interviewers supervised by Project staff	Jan 2012 and June 2015	EPI INFO and Excel data bases
Coverage of childhood immunization interventions	Measles immunization among children 12- < 24 months Complete vaccination coverage among children 12- < 24 months (BCG, PENTA 1–3, polio 1–3, measles)	KPC surveys	Contracted interviewers supervised by Project staff	Jan 2012 and June 2015	EPI INFO and Excel data bases
Coverage of WASH (water, sanitation and hygiene) interventions	Regular point-of-use water treatment Safe water storage Safe disposal of child’s feces following most recent defecation Appropriate hand-washing station in the home (with soap, water, and water container) Appropriate handwashing (at 4 critical moments)	KPC surveys	Contracted interviewers supervised by Project staff	Jan 2012 and June 2015	EPI INFO and Excel data bases
Birthing Center activities	Number of services provided Number and types of birth complications that arose and their management	Birthing Center clinical records	Birthing Center supervisory and auxiliary nurses	Monthly	Excel database for Birthing Center services
Extension of Coverage Program (PEC) outputs	Same output and outcome indicators as for the Project	Extension of Coverage Program (PEC) data collection forms from national health management information system	PEC ambulatory nurses and facilitators	Monthly	National Health Management Information System and *Sistema de Información Gerencial de Salud* (SIGSA)
Women’s and community empowerment and social capital indicators	Women’s decision-making autonomy concerning place of delivery, child treatment for pneumonia, and use of family planning Women’s control of family money for purchase of food and healthcare for children Community execution of projects to benefit community well-being Number of communities with an emergency transport system for women in labor or for ill children Women’s participation in Care Groups Women’s participation in community meetings	KPC surveys	Contracted interviewers supervised by Project staff	Jan 2012 and June 2015	EPI INFO and Excel data bases
Demographic characteristics of *comadronas*	Age, gender, level of education, literacy, number of years as *comadrona,* how trained, number of deliveries attended	Census of *comadronas*	Level-2 Promoters supervised by graduate student intern	July 2013	Excel data file
Effectiveness of CBIO+ Approach	Opinions, observations and recommendations of Project field staff and MSPAS staff	FGDs, group interviews, key-informant interviews, and structured questionnaires with Project field staff, and MSPAS staff	Curamericas Global staff Graduate student interns Level-2 Promoters	Aug 2013 May–June 2015	Transcriptions of recordings, completed questionnaires and Excel/Word analysis tables
Integration of c*omadronas* into Birthing Centers	Opinions, observations, and recommendations of *comadronas* regarding their participation with the Birthing Centers	Key-informant interviews	Level-2 Promoters Graduate student interns Birthing Center staff	Aug 2013 June 2015	Transcriptions of recordings and Excel/Word analysis tables
Empowerment of women by Project and by Care Group Approach	Opinions, observations and recommendations of women, husbands, community health committees, mothers-in-law Opinions, observations and recommendations of women participating in Care Group approach	FGDs with husbands, community health committee, mothers-in-law FGDs with Care Group Volunteers and women participants in Self-Help Groups Key-informant interviews with Level-1 Promoters	Level-2 Promoters (January 2014) Contracted interviewers (May 2015)	Jan 2014 (women’s empowerment as a result of the Project) May 2015 (empowerment as a result of the Care Group Approach)	Transcriptions of recordings and Excel/Word analysis tables
Birthing Center clinical quality of care	Outcomes of complications managed by the Birthing Centers Opinions, observations and recommendations of Birthing Center staff and Curamericas/ Guatemala Project Director with respect to management of perinatal complications	Birthing Center clinical records Structured written questionnaire FGDs with staff Key-informant interview (with Project Director)	Curamericas Global staff Graduate student interns	Dec 2016	Excel spreadsheets; Completed questionnaires; Transcriptions of FGDs and key- informant interviews and Excel/Word analysis tables

“Birthing Center” refers to Community Birthing Centers *(Casas Maternas Rurales*); *FGD* Focus group discussion; *DALY* Disability-adjusted life year

### The process of data collection

Baseline knowledge, practice, and coverage (KPC) household surveys were used to establish quantitative baseline measures for intervention coverage and child nutritional status, as well as baseline measures for empowerment indicators.

During the first two years of the Project (Phase 1), formative research was conducted to (1) assess and document the challenges and advantages of implementing the CBIO+ Approach and integrating it within the MSPAS framework for health care delivery, (2) establish and assess a new role for *comadronas* (traditional birth attendants) in maternity care, and (3) measure constructs such as community engagement and women’s empowerment. Methods of data collection included focus group discussions (FGDs), group interviews, and key-informant/in-depth interviews, all with informants who included women of reproductive age, Care Group Volunteers (*Comunicadoras*), men/husbands, community leaders, *comadronas*, and staff of both Curamericas/Guatemala and MSPAS.

During the first two years of the Project (Phase 1), Area A constituted the intervention study area, and Area B constituted the Phase 1 comparison study area. In Phase 2, Area B continued to serve as a quasi-comparison area based on the hypothesis that longer exposure to Project interventions and the CBIO+﻿ Approach in Area A would result in superior outcomes relative to Area B because of a dose–response effect. Anthropometric monitoring of all children younger than 2 years of age (hereafter referred to as under-2 children) and analyses of Vital Events Registers were conducted on an ongoing basis to monitor changes in nutrition indicators and in maternal and child mortality.

An endline KPC survey and final analysis of the Project’s Vital Events Registers were used to examine results in relation to the Project’s initial hypotheses and implementation research questions. Endline qualitative research also explored (1) an assessment of the challenges and advantages of implementing the CBIO+ Approach, and (2) an assessment of the effect of women’s participation in the Care Group training cascade on their self-efficacy and autonomy.

#### Quantitative methods

##### Household KPC surveys

Standard modules [13] were used to measure the baseline and endline outcome indicators in the KPC surveys. The survey covered the complete set of health and women’s/community empowerment indicators and was carried out independently in Areas A and B in January 2012 and again independently in Areas A and B in June of 2015. Informants were mothers of under-2 children who were in 30 randomly selected clusters and in 10 randomly-selected households in each of the clusters. Distinct communities/villages functioned as clusters grouped according to size, so that each separate household survey was designed to have a total of 300 respondents for each Area. (The January 2012 baseline KPC for Area A had 299 informants because one informant was later found to be ineligible.)

The structured questionnaire was written first in English, then translated into Spanish by an external evaluator and Curamericas/Guatemala staff. Interviewers who were native speakers of the local Maya dialect then translated the Spanish into the local Maya dialect (which is not written) at the time of the interview after having first come to a consensus during their training for the optimal translation. Mothers were interviewed in their households using the Maya language by bilingual (Spanish/Maya) interviewers. Interviewers were Maya women with at least a high-school-level education who were hired and trained specifically to administer the survey. They received four days of training. The training included an explanation of the Project’s goals and indicators, interviewing skills, anthropometry skills, oral translation of the Spanish questionnaire into the three local Maya languages, and field practice with interviewer skills observed and evaluated by Curamericas/Guatemala interviewer supervisors.

Excel spreadsheets were used to tabulate and analyze baseline survey results and generate values and confidence intervals for each result from each Area. All Excel data entries were cross-checked by the tabulators, who were trained Curamericas/Guatemala staff. A year later, the baseline Excel dataset was entered into Epi Info 7 and the initial results confirmed by a graduate student intern. Endline Project data were entered into Epi Info 7 by trained Curamericas/Guatemala tabulators who performed cross-checking of all data entry. Epi Info 7 was then used to obtain lists, frequencies, and tables that included calculated percentages, means, medians, and ranges for all indicators and demographic data points, as well as confidence intervals/margins of error for each proportional result. Statistical significance was determined by obtaining *p*-values comparing differences for the same indicator for the baseline and final KPC survey in each Area using Epi Info Stat Calc. All *p*-values < 0.05 were reported as statistically significant. All KPC survey data are available online [14, 15].

##### Childhood anthropometry household surveys and censuses

Anthropometric data for under-2 children were collected from three household surveys:

1. The Baseline KPC Survey of 599 mothers of under-2 children conducted in January 2012 in 30 Area A communities (*n* = 299) and 30 Area B communities (*n* = 300), as described above. These surveys collected only weight and not height of the youngest under-2 child in the household. Only underweight was calculated.
2. In September 2012, 288 mothers of under-2 children from 30 Area A communities. The height as well as weight of each of the mothers’ youngest under-2 child was measured, enabling the calculation of stunting, underweight, and wasting.
3. As part of the Final KPC Survey in June 2015, for which 300 mothers of under-2 children in 30 Area A communities and 300 mothers of under-2 children in 30 Area B communities. The height and weight of each of the mothers’ youngest under-2 child was measured, again enabling the calculation of stunting, underweight, and wasting.

The specially trained contracted interviewers who carried out the January 2012 and June 2015 KPC surveys also executed the anthropometry for those surveys while Level-2 Promoters were trained to perform the anthropometry for the September 2012 survey. Interviewers’ anthropometry skills were verified by training supervisors during field practice.

The anthropometric data were first analyzed with Epi Info 7 using z-scores to detect and eliminate outliers (i.e., those scores that were less than or greater than 6 standard deviation units from the reference mean). A z score is the value obtained after converting all of the actual scores into a distribution that has a mean of zero, with the z score indicating the number of standard deviation units above or below the mean. The data sets for each survey, without outliers, were then exported into Excel tables, where each entry was reviewed for correct classification and corrected as necessary using the WHO reference tables for underweight, stunting, and wasting. This was followed by counting of the records with children who were underweight, stunted, and wasted (i.e., z-scores < -2SD) and calculating undernutrition prevalence. Two separate independent researchers corroborated these results. All *p*-values (Fisher mid-point) were calculated for comparisons using WinPepi [16].

Apart from these anthropometric surveys, the Project also conducted anthropometry at other times for all children. Beginning in June 2013 in Area A communities and in August 2014 in Area B communities, all under-2 children were weighed at least twice per year. These were in essence anthropometric censuses of under-2 children, since 93–100% of these children were weighed at the time of each survey. The final anthropometric censuses were conducted in November 2014 in both Area A and B. Level-2 Promoters, assisted by Level-1 Promoters, weighed and measured every under-2 child in their assigned communities during a home visit and utilized the WHO weight-for-age (WFA), height-for-age (HFA), and weight-for-height (WFH) reference tables to identify all children who were underweight, stunted or wasted (< -2SD). The classifications were checked independently by Curamericas/Guatemala M&E staff who then transferred this data to Excel spreadsheets and aggregated the data by Area and by municipality (district). WinPepi was used to calculate *p*-values (Upton's “N—1” chi-square) for all of these comparisons.

##### Quality of clinical care data at Community Birthing Centers (*Casas Maternal Rurales*)

The Birthing Center*s* maintained clinical records which included data on complications of individual patients during the prenatal period, during labor and delivery, and during the postpartum period. Paper 6 [6], on management of clinical complications in the Birthing Centers, is based on an analysis of patient records for the Birthing Centers in Calhuitz (2009–2016), Santo Domingo (2013–2016) and Tuzlaj Coya (2014–2016). Complication registers, which noted every complication captured in the clinical records of the three Birthing Centers*,* were created by the Curamericas Global investigators and graduate student interns using Microsoft Excel. Complications tracked include complications immediately before, during, and immediately after birth (peripartum complications). The analysis incorporated cumulative registry data from the initiation of each of the three Birthing Centers through June 2016 (Table 2).

**Table 2:**
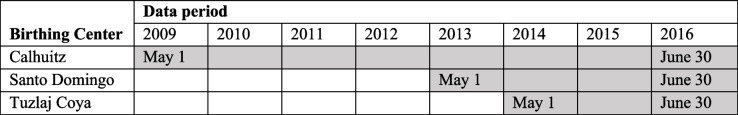
Registry data collection period at Community Birthing Centers (*Casas Maternas Rurales*)

The registers included non-identifying demographic information on clients, their condition and the care received; whether the complication was resolved in the Birthing Center or the mother was referred to a hospital; and, in the case of referrals, the name of the referral facility, the services provided there, and the outcome for the mother. A descriptive analysis of register data utilizing Microsoft Excel data tables was performed to answer the quantitative research questions.

#### Qualitative methods

##### Focus group discussions

Members of the implementation research team led the focus group discussions (FGDs). In all cases, 5–8 respondents were selected in order to provide a reasonable representation of the study population. FGDs were carried out for a number of different evaluation activities. FGDs were conducted by well-trained teams of three or four investigators, usually Level-2 Promoters who were bilingual native speakers of Spanish and the local Maya language. Teams included (1) a leader who asked the questions, (2) one or two recorders who took notes, and (3) a timekeeper who also managed the recording if the FGD discussion was being recorded. FGDs with Project beneficiaries and family members were conducted in the local Maya language, and FGDs with Project and MSPAS staff were conducted in Spanish. Verbal informed consent was obtained from all participants, and FGDs were held in spaces that allowed for privacy. No personal identifying information was recorded or transcribed. The notes and/or recordings were translated and transcribed into Spanish MSWord documents by either the FGD team members or by the lead research team investigator. Analysis of the written transcriptions was done by research team investigators utilizing a variety of deductive and inductive methods, as appropriate for the subject matter. Further details about specific FGDs are indicated below.

1. Assessment of clinical quality of care for complications at Community Birthing Centers (Paper 6 [6])
  A FGD was held in December 2016 that included the three Supervisory Nurses for the three Birthing Centers, the auxiliary nurse at one of the Birthing Centers and two support women (each from a different Birthing Center). This FGD was designed and led by a bilingual Curamericas Global staff member (BM). Topics included how the decision was made to treat or refer complications, why families refused referrals and the effects of those refusals, the complications that women from partner communities presented with compared to those of women from non-partner communities, coordination of services with MSPAS, and the vision for the future of the Birthing Centers and the services that they might be able to provide.
  The FGD was held remotely via Skype; conducted, recorded and transcribed in Spanish; and translated to English by the Curamericas Global staff member. The English transcription was then analyzed by two investigators using Microsoft Excel and Microsoft Word. Responses were coded inductively using systematic, thematic coding. All responses were matched to a predetermined codebook. New codes were created for additional themes as needed. The findings served to complement the quantitative data for clinical care at the Birthing Centers described earlier.
2. Assessment of women’s empowerment (Paper 8 [8])
  In January 2014, 17 FGDs were conducted with mothers of under-2 children, men/husbands, community health committees, and mothers-in-law (*suegras*) from 13 randomly-selected communities drawn from only Area A communities of all three municipalities. Each group consisted of only one class of informant. All were gender-specific except the groups for the health committees, which included men and women. The primary purpose was first to explore how the Project had improved the status, decision-making autonomy, and agency of women. Secondly, the purpose was to explore the local facilitators and barriers to women’s empowerment. The FGDs were conducted by Level-2 Promoters in the local Maya languages. These FGDs were not recorded; instead, two bilingual note-takers for each FGD took notes in Spanish, paraphrasing key statements and occasional direct quotes. The notes were then transferred in Spanish into a Microsoft Word document. A bilingual English/Spanish-speaking Curamericas Global staff member on the research team performed an analysis of the transcriptions, which had been entered into thematically-organized Excel tables. The analysis used both Grounded Theory [17] and codification based on the identification of the specific facilitators and impediments to women’s empowerment. Substantive coding was used to identify themes and concepts, and axial coding was used to combine them into macro-concepts/themes and to identify associative and possible causal links.
3. Overall assessment by staff and key stakeholders of the CBIO+ Approach (Paper 9) [9]
  Two FGDs were conducted with key personnel in August 2013. The purpose was to assess their knowledge of the CBIO+ Approach and hear their perspectives on its strengths and weaknesses as well as ways it could be improved. The FGDs were held with (1) four MSPAS employees providing services to the San Sebastián Coatán municipality through the *Sistema Integral de Atención en Salud* (SIAS), a program of the MSPAS that coordinated with the Project; and (2) six Curamericas/Guatemala Level-2 Promoters from San Sebastián Coatán. The FGD with SIAS staff included: two health educators, one nurse and a health information specialist. Because this group did not include any Curamericas/Guatemala Project staff and because these MSPAS employees had not been exposed to any information specifically about the CBIO+ Approach, the FGD focused on the relationship between SIAS and Curamericas/Guatemala and opportunities for enhanced collaboration. The FGD with the six Level-2 Promoters from San Sebastián Coatán municipality included four who were familiar with the CBIO+ Approach and two who had been recently hired. The FGD explored their perspectives on the strengths and weaknesses of the CBIO+ Approach and recommendations for its improvement.
  The FGDs were conducted in Spanish and led by a Spanish-speaking graduate student intern using a list of questions as a guide [18] and a tape recorder. The recordings were transcribed into Spanish by the intern and then analyzed by the intern by coding the responses into thematic categories based on the research questions.

##### Group interviews

We use the term “group interviews” here to distinguish them from FGDs. With the group interviews, three to eight participants responded to straightforward questions posed by the interviewer without trying to stimulate further discussion or interchange of ideas between participants. Group interviews, rather than key-informant/in-depth interviews, were used when (1) the subject matter was not sensitive or highly personal, (2) time or research staff constraints precluded conducting individual interviews, and/or (3) available research staff did not possess the skills required to properly facilitate an FGD. Otherwise, the procedures followed were the same as those for FGDs described above.

For Paper 7 in this supplement [7] on community empowerment and the effect of the Care Group approach on the social status, self-efficacy, decision-making autonomy, and social capital of its female participants, group interviews (as opposed to FGDs) were carried out with Care Group Volunteers and Self-Help Group participants by teams of trained contracted interviewers. The purpose was to assess (1) if the Care Group approach empowered and increased the status and agency of the participants and generated community social capital and (2) the Project's implementation of the Care Group Approach and elicit suggestions for its improvement. These group interviews took place in the communities of Ququilum and Jajhuitz in the municipality of San Sebastian Coatán, in Paiconop Grande and Aldea Poza in the San Miguel Acatán municipality, and in Altamiranda and Kanajaw Xixilack in the Santa Eulalia municipality. Curamericas/Guatemala staff chose these communities because they were readily accessible and were considered representative of the Care Group experience in each municipality.

Three teams each consisting of three interviewers with at least secondary-level education, native-language speaking ability in the local Maya language, and fluency in Spanish were hired from each of the three municipalities to carry out these group interviews. One of the authors (CG) trained these nine interviewers in the methods of in-depth and group interviews, as well as in the purpose of the Project and its implementation research, the fundamentals of qualitative evaluation, and the content of the interviews. The interview questions had been previously translated from English to Spanish by a team of two bilingual native English speakers and three Guatemalan native Spanish speakers. The interviewers then collaboratively translated each interview question from Spanish into the local Maya languages in use in the Project Area (Chuj, Akateko, Q’anjob’al).

The group interviews were conducted during the program’s final evaluation in May 2015 in the local Maya language of each municipality. One interviewer asked questions, one wrote down the responses in Spanish, and a third noted behaviors in the group and verified the transcription of the responses. To reduce the potential for bias, interview team members rotated among the roles of interviewer, secretary/transcriber, and observer.

In each selected community, all the Care Group Volunteers and all members of a randomly selected Self-Help Group were interviewed. At the time of the meeting in the selected community, 6–8 Care Group Volunteers in the community were interviewed along with 8–9 women in each Self-Help Group. Thus, a total of six groups with 6–8 Care Group Volunteers in each, and six groups of 8–9 Self-Help Group members, providing a robust representation, participated in these interviews. The notes of each group interview were transcribed into Spanish by the interview teams. The Spanish transcripts and the observational notes from the interviews were translated into English for evaluation and analysis by a bilingual program evaluator. The English transcript content was organized into Excel files and analyzed using deductive thematic analysis focused on four social constructs: perceived social status, self-efficacy, decision-making autonomy, and formation of social capital.

For Paper 9, on the evaluation of the strengths and weaknesses of the combined CBIO+ Approach [9], as a follow-up to the August 2013 investigation of these themes, group interviews were conducted in June 2015 with Curamericas/Guatemala Project staff and with MSPAS staff familiar with the CBIO+ Approach. Interviewees were selected from all three of the Project’s municipalities: San Sebastián Coatán, San Miguel Acatán, and Santa Eulalia. There was equal representation of Level-2 Promoters from each of the three municipalities (*n* = 7, 7, and 7, respectively), and near equal representation of MSPAS staff from each of the three municipalities (*n* = 3, 5, and 3, respectively). The MSPAS staff interviewed included five auxiliary nurses, three professional nurses, a doctor, a secretary, and a counselor.

The Level-2 Promoters were interviewed in Spanish in small groups of two or three by a Curamericas/Global graduate student intern. MSPAS staff from San Sebastián Coatán and San Miguel Coatán were interviewed by the same investigator in small groups of two to four. The MSPAS staff were selected based on availability. The three MSPAS staff from Santa Eulalia were not available to be interviewed in person and instead completed the interview in writing utilizing the same questionnaire used for the group interview. Also, due to logistical issues, the seven Level-2 Promoters from San Sebastián Coatán, an MSPAS Auxiliary Nurse also from San Sebastián Coatán, and an MSPAS Counselor from San Miguel Acatán were interviewed utilizing the same group questionnaire individually rather than in a small group.

Separate sets of interview questions were created for the Level-2 Promoters and for the MSPAS staff. The interview questions were developed in English, translated to Spanish, then back-translated to English for validity, and finally administered in Spanish. A graduate student intern (a bilingual, native English speaker) conducted the interviews. The intern transcribed the interview responses in Spanish in real time and then translated the transcriptions from Spanish to English for analysis. Audio recordings were also made for reference. The themes from the updated Community Health Worker Assessment and Improvement  (CHW AIM) Toolkit were applied in the analysis of the data.

##### Key-informant interviews

We used key-informant interviews to obtain answers to specific questions. Higher-level staff members as well as lower-level staff (Level-1 Promoters) members participated in these*.* We used this format, rather than a FGD or group interview format, when it was not feasible to meet with the respondents as a group, when the subject matter was considered sufficiently sensitive to render it less advisable to discuss it in a group, or when (in the case of the interview with the Project Director) there was no other person at his/her level to interview.

For the assessment of the Care Group approach’s impact on women’s empowerment, perceived social status, agency and social capital (Paper 7 [7]), in-depth interviews were conducted with the Level-1 Promoter of each of the following six communities: Ququilum and Jajhuitz in the municipality of San Sebastian Coatán, Paiconop Grande and Aldea Poza in the San Miguel Acatán district, and Altamiranda and Kanajaw Xixilack in the Santa Eulalia district. The Curamericas/Guatemala staff chose these communities because they were representative of the Care Group experience in each municipality, and the communities were also readily accessible. Each community had its own Level-1 Promoter.

For these interviews, three interviewers with at least a secondary-level education, native speaking ability in the local Maya language, and fluency in Spanish were hired from each of the three municipalities represented in the study. These nine interviewers were trained by the field investigator leading the study in the methods of in-depth and group interviews as well as in the purpose of the Project, the fundamentals of qualitative evaluation, and the content of the interviews. The interviews followed a structured questionnaire whose questions had been previously translated from English to Spanish by a team of two bilingual native English speakers and three Guatemalan native Spanish speakers. The interviewers then collaboratively translated each interview question from Spanish into the local Maya language. Nine interview questions and 21 follow-up questions were designed to elicit information necessary to answer the three primary research questions mentioned above.

The Spanish transcripts and notes from the interview were translated into English for evaluation and analysis by a bilingual program evaluator. Following translation to English, the data were analyzed for themes using a combination of open and axial coding [19].

For Paper 6 [6] on the clinical quality of care provided at the Birthing Centers with respect to the management of complications and issues of family compliance or non-compliance with referrals for hospital care, one key-informant interview was held in December 2016 with the Project Director (MV) by the lead investigator (a Curamericas Global staff member). The Project Director had been intentionally left out of the FGD to encourage Birthing Center staff to speak openly about their experiences. In addition, he possessed deep knowledge of the social dynamics and culture of the local Maya population. The interview was conducted over Skype in Spanish and recorded. The recording was transcribed first in Spanish and then translated to English by the bilingual lead investigator. The transcription was then analyzed by two investigators using Microsoft Excel and Microsoft Word. Responses were coded inductively using systematic, thematic coding. All responses were matched to a predetermined codebook, with new codes created for additional themes as they emerged.

For Paper 9 [9] on the overall effectiveness of the CBIO+ Approach, key informant interviews took place in August 2013, and these interviews were designed as a follow-up to an earlier self-administered questionnaire and to gather more information from four key Curamericas/Guatemala staff members possessing unique perspectives on the CBIO+ Approach and the Project: the Municipal Coordinator and the Institutional Facilitator for San Sebastián Coatán, a Level-1 Promoter from San Sebastián Coatán, and the Project M&E Specialist. Due to logistical challenges, it was not possible to interview individuals from beyond the San Sebastián Coatán municipality. The interviews were designed and carried out in Spanish by a Spanish-speaking graduate student intern.

##### Self-administered questionnaires

Self-administered open-ended questionnaires in Spanish MSWord documents were occasionally used with informants from Curamericas/Guatemala and MSPAS staff. This strategy was chosen to allow them time for individual reflection concerning topics of a technical nature (e.g., details of the CBIO+ Approach, the approach to managing perinatal complications in the Birthing Centers). Those completing the questionnaire later either received a follow-up interview or participated in an FGD. In both cases, the questions that were posed had been developed from the responses to the completed questionnaire. Completed questionnaires were either hand-written on a print-out of the questionnaire or completed electronically and emailed by informants as a MSWord document to the lead investigator.

For the assessment of issues related to clinical care of perinatal complications provided at Birthing Centers*,* described in Paper 6 [6], 12 Birthing Center staff members at all levels (supervisory nurses, auxiliary nurses, and support women) at three separate Birthing Centers completed a self-administered questionnaire in November 2016. The questionnaires were Word documents that were received by email from a Curamericas Global staff investigator. The completed questionnaires were not anonymous but were kept confidential, with only the investigators having access to them. The responses to the questionnaire guided the drafting of the questions for the FGD that followed soon afterward.

For the assessment of the overall effectiveness of the CBIO+ Approach described in Paper 9 [9], a hand-written paper-and-pencil self-administered questionnaire was given in July 2013 to all Curamericas/Guatemala staff from all three municipalities to complete. The questionnaire focused on several key areas: (1) staff knowledge of the key CBIO+ elements; (2) staff perceptions of the major advantages, disadvantages and challenges of the approach; and (3) ways that the CBIO+ Approach could be improved.

The Project utilized two different versions of this self-administered questionnaire to explore each staff member’s perspective on the strengths and weaknesses of the CBIO+ Approach. The first version was given to the Project’s three Municipal Coordinators to complete individually to explore their perspectives on the CBIO+ Approach and to receive feedback on the self-administered questionnaire quality so that improvements could be made before it was distributed to the remainder of the staff.

This first version had 19 questions that covered each of four principal areas of interest. Based on the input from the Municipal Coordinators,  the questionnaire was revised and sent to the remainder of the staff. The second version of the self-administered questionnaire consisted of 23 questions that covered each of three major areas of investigation and was distributed to all Project personnel in August 2013. Twenty-one people took the second version of the survey, including two Municipal Coordinators, the M&E Assistant, and 18 Level-2 Promoters. The results were then coded, tabulated, and analyzed.

##### Vital events registration

For our implementation research, vital events were defined as newly identified pregnancies, births (both live births and stillbirths), and deaths. The source of data analyzed was the Project’s Vital Events Registers. These were Excel files maintained by the Project’s three Institutional Facilitators, one for each municipality. Each of the Institutional Facilitators was a Registered Nurse trained in the CBIO+﻿ Approach and in the conduct of vital events registration and verbal autopsies. For each of the three municipalities there were two Vital Events Registers, one with the vital events data from the Area A communities in that municipality and the other with the vital events data from the Area B communities in that municipality. Thus, there were six Registers in all, each with its own Excel file. There were four spreadsheets in each Register, each containing a specific data set: (1) pregnancies and pregnancy outcomes (stillbirths and live births); (2) under-5 deaths including the findings from the verbal autopsies – described further below; (3) deaths among women of reproductive age, with a notation of whether it was a maternal death (related to pregnancy, delivery, or during the 6-week-postpartum period) and also including the findings from the verbal autopsy; and (4) a general mortality registry including data for deaths of older children, men, and women who were not of reproductive age.

Every new pregnancy, live birth, stillbirth, under-5 death, and maternal death had a unique 12-digit identification number that prevented duplication of data and that enabled the location of specific vital events in the Vital Events Register utilizing the data sorting/filtering capacity of Excel. The identification number was constructed using a standardized method that utilized code numbers that captured which Area the community was in (Area A or Area B), as well as the municipality, community, name of the Level-2 Promoter for the community in which the subject lived, and identification number of the pregnancy. The pregnancy was also later further specified as resulting in a live birth or a stillbirth. If a live-born child later died, this death was also given an identification number.

The pregnancy/pregnancy outcome register included the mother’s name, residence, date of birth, age, due date, actual delivery date and delivery outcome, including if the outcome was a stillbirth. The under-5 mortality register included the child’s name, date of birth, mother’s name and residence, mother’s age and date of birth, date of child’s death, age group of the child at the time of death (neonatal, 1-<12 month, 12-<60 month), age in days at death for neonatal deaths, primary and secondary causes of death, which of the four delays (described further in Appendix 1) contributed to the death, place of death, and, for neonatal deaths, place of delivery. Notes from the verbal autopsy elucidating the contributing factors were also included. The maternal death register included the mother’s name, residence, date of birth, age at death, and, for maternal deaths, cause of death (primary and secondary), place of death, place of delivery, and verbal autopsy notes. The general death register (for all other deaths) tracked data similar to the maternal death register.

The vital events data were collected by a Level-1 Promoter (all of whom were female) in each community every two weeks at a meeting with the Care Group Volunteers she was training and supervising. The Care Group Volunteers kept track of 10-15 of their women neighbors who were mothers of under-2 children with whom they met every two weeks to share lessons on health behaviors and to collect vital events. Collectively, the Care Group Volunteers kept track of the vital events of every family in which there was a mother of an under-2 child. In addition, they also detected and reported to their Level-1 Promoter any vital events that occurred in other households in their community, providing for a broader surveillance for vital events. The Level-1 Promoter in turn reported this information to the Level-2 Promoter who met with the Level-1 Promoter twice a month for training on how to guide the Care Group Volunteers in teaching their lessons and in collecting the vital events data the Level-1 Promoters had gathered from their Care Group Volunteers.

The Level-2 Promoter collected these data from the 5–8 Level-1 Promoters she supported in her assigned communities. This was supplemented by vital events detected by the Level-2 Promoters during their home visits and during their monthly meetings with the Community Health Committees. In addition, the local *comadronas* would inform both Level-1 and Level-2 Promoters of vital events they had detected. The Level-2 Promoter passed the vital events data to his/her Care Group Supervisor, who collated that municipality’s vital events data received from all of the 5–10 Level-2 Promoters in that municipality and then conveyed those data to the Institutional Facilitator for her municipality. The collated municipal vital events data were recorded by the Municipal Institutional Facilitator in the municipal Vital Events Registers for Area A and for Area B. For each maternal and child death, the Municipal Institutional Facilitator conducted a verbal autopsy to identify the cause of death and contributing factors (see below).

Every month, each of the Institutional Facilitators sent the updated Vital Events Registers for their municipality to the Institutional Facilitator Supervisor who was based at the Project headquarters. The Institutional Facilitator Supervisor first reviewed the registers for data integrity, then used the cleaned registers to update sets of Excel tables that computed the following data for each Area for each municipality: (1) birth rate, (2) neonatal mortality rate, (3) 1-<12 month (post-neonatal) mortality rate, (4) 12-<60 month mortality rate, (5) infant and under-5 mortality rates, (6) aggregated causes of death for all three child age groups, (7) maternal mortality ratio, and (8) aggregated causes of maternal deaths. The Project reviewed these results every quarter.

For the mortality assessment study (Paper 5 [5]), an investigator hired for the Project’s final evaluation (SB) worked with the Institutional Facilitators to finalize the Vital Events Registers. This involved identifying missing verbal autopsies, conducting those autopsies, and adding their data to the Registers. Once the Registers were cleaned, a Curamericas Global staff member of the research team reviewed and entered the Register data into the Excel tables created by the Institutional Facilitator Supervisor. New tables, similarly organized by municipality and Area, were created that included (1) distribution of ages at death (in days) for neonatal deaths during the first 28 days of life, (2) perinatal mortality rate (stillbirths and early neonatal deaths), (3) distribution by age groups of under-5 deaths, (4) distribution over time of the four delays for under-5 deaths, (5) distribution over time of the four delays for maternal deaths, (6) place of death for under-5 deaths, (7) place of death for maternal deaths, and (8) place of delivery for maternal and for neonatal deaths. In addition, a parallel dataset using the same tables was created for just the 26 partner communities of the three active Birthing Centers in order to assess trends in maternal and neonatal mortality in these communities. These Excel tables provided the data used for the analysis of the Vital Events reported in Paper 5 [5].

##### Verbal autopsies

When the Vital Events Register contained a report of a maternal or child death, the Institutional Facilitator followed up, within two weeks if possible, by performing a verbal autopsy with the family of the deceased woman or child. The information obtained in the Vital Events Register contained all the information the Institutional Facilitator needed to locate the family with the aid of the community’s Level-1 Promoter (i.e., name of the deceased, date of death, name of community, and the names of the Care Group Volunteer, Level-1 Promoter, and Level-2 Promoter) so the path of the data flow could be tracked to facilitate any data cleaning that might be needed.

The Institutional Facilitator completed an MSPAS standard verbal autopsy form [20] by hand. The most salient information from the verbal autopsy was added to the electronic Vital Events Register. Thus, the Vital Events Register had for each death the following information for each death: date of death, birth date (for children dying before reaching 5 years of age); age group for under-5 deaths – neonatal, post-neonatal, or 11–59 months; age at death (in days for neonates, in months for post-neonates); classification of the cause of death (the process for this is described in Appendix 1); location of death; location of delivery (for maternal and neonatal deaths); which of the “four delays” contributed to the death (also described in Appendix 1); and notes that included a brief narrative of the circumstances of the death, including whether treatment was sought and, if treatment was obtained, by whom, when, and how the treatment was obtained; and if no treatment was sought or if there was a delay in seeking treatment, the family’s stated reason for this. The information obtained in the verbal autopsy also enabled the Institutional Facilitator to distinguish stillbirths from neonatal deaths as well as to distinguish maternal deaths from non-maternal deaths among women of reproductive age.

##### Cost analysis

One of the purposes of Paper 10 [10] was to summarize the costs incurred by Curamericas Global and Curamericas/Guatemala in implementing the Project from October 2011 through September 2015 and to calculate the annual costs of the Project, including the costs of the Birthing Centers, on a per beneficiary basis (women of reproductive age and under-5 children) and on a per capita basis (using the entire population of all age groups and sexes). Part of the reason for this exercise was to determine the feasibility of adoption of this approach by the Government of Guatemala and whether the local municipal governments might have the capacity for long-term sustainability of the CBIO+ model.

A substantial portion of Project activity funds were from the United States Agency for International Development’s Child Survival and Health Grants Program, which supported the community-based child survival activities, and from the Ronald McDonald House Charities, which supported the Birthing Center program. For calculation of program costs, we used only the in-country Guatemala expenses ($1,515,075), which accounted for 75.6% of the total funds that were available to operate the Project. We also calculated the population served and the number of beneficiaries from census data collected by the Project. We then divided the total costs by the population size as well as by the number of beneficiaries to calculate the annual cost per capita and per beneficiary, respectively, for each year of the Project. With this information, along with the mortality data from Paper 5 [5] we calculated the cost-per-life saved and cost per disability-adjusted-life-year (DALY) averted.

### Summary of methods

Table 3 contains the implementation details for data collection and presents for each research question (1) the data collection methods used, (2) the sampling method, (3) who the participants/study subjects were, (4) when and where the investigation was done, and (5) the product of the investigation and where the final report for that topic can be found. The implementation research utilized both quantitative and qualitative methods, attempting whenever feasible to triangulate quantitative findings with qualitative findings.

**﻿Table 3:** Data collected for testing specific research questions

Hypotheses/research questions	Data collection methods	Sampling methods	Informants/ data sources	Dates/locations
The CBIO+ Approach improves the population coverage of interventions that are designed to address the epidemiological priorities for mothers and children relative to a Comparison Area (Area B) and compared to (1) baseline levels, (2) selected nearby municipalities of the Department of Huehuetenango where the Project was not working (using data obtained in MSPAS documents), and (3) the rural population of the Department of Huehuetenango (using data from the national Demographic and Health Survey). Findings presented in Paper 3 [3].	Baseline KPC survey	30-cluster stratified cluster sampling	300 mothers of under-2 children in each Area	January 2012/ both Areas A and B
	Endline Project KPC survey	30-cluster stratified cluster sampling	300 mothers of under-2 children in each Area	June 2015/ both Areas A and B
The CBIO+ Approach improves the nutritional status of children relative to a Comparison Area (Area B) and relative to (1) baseline levels, (2) selected nearby municipalities of the Department of Huehuetenango where the Project was not working (using data obtained in MSPAS documents), and (3) the rural population of the Department of Huehuetenango (using data from the national Demographic and Health Survey). Findings are presented in Paper 4 [4]	Baseline KPC survey	30-cluster stratified cluster sampling	300 under-2 children weighed in both Areas A and B	January 2012/ both Areas A and B (weight but not height measured)
	Anthropometric household survey	30-cluster stratified cluster sampling	288 under-2 children weighed and measured	September 2012/ Area A only
	Anthropometric “censuses”	No sampling since all children had their weight and height measured (by visiting all homes)	All under-2 children weighed and measured (ranged from 871 to 1,203 children in each “census”)	June﻿ 2013, Sept 2013, and January 2014 (Area A only) Aug 2014 and Nov 2014 (both Areas A and B)
	Endline KPC survey	30-cluster stratified cluster sampling	300 under-2 children weighed and measured for each Area	June 2015/ Both Areas A and B
The CBIO+ Approach reduces under-5 and maternal mortality relative to a Comparison Area (Area B) and compared to (1) baseline levels, (2) selected municipalities of the Department of Huehuetenango where the Project was not working, and (3) the rural population of the Department of Huehuetenango. Findings presented in Paper 5 [5].	Gathering of all vital events in Project Area	Analysis of Register data for all births, stillbirths, and maternal/U-5 deaths	Vital events gathered by *Comunicadoras,* Level-1 Promoters, and Level-2 Promoters	Oct 2011 to May 2015 (Area A) Oct 2013 to May 2015 (Areas A and B)
	Verbal autopsies	Analysis of verbal autopsies for all maternal and U-5 deaths	Families of 34 deceased women and 314 under-5 children	
	MSPAS mortality data for the Department of Huehuetenango	Analysis of MSPAS maternal and under-5 mortality data for the Project’s 3 municipalities and for 3 comparison municipalities outside of the Project Area	Government national vital events registry﻿ (*Registro Civil*)	July 2015 (3 comparison municipalities outside the Project Area)
The staff of the Birthing Centers successfully managed many perinatal complications and in the process contributed to the reduction in maternal and perinatal mortality in the Project communities over the course of the Project. The success of the Birthing Center staff in managing complications can be attributed to intensive training and an extensive support network The management of complications is often compromised by delays in reaching the Birthing Center or by not accepting referral from the Birthing Center staff when recommended Findings are presented in Paper 6 [6]	Review of clinical records	Clinical records of deliveries with complications	Register created of complications recorded in clinical records	July–Aug 2016
	Self-directed written questionnaire	Purposive sample of staff	Birthing Center staff: Supervisory Nurses, Auxiliary Nurses, and support women	Nov 2016
	FGDs			Dec 2016
	Key-informant interviews	Purposive sample of staff	Project Director	Dec 2016
The CBIO+ Approach produces significant increases in women’s participation in community health activities relative to the Comparison Area. The CBIO+ Approach produces significant increases in women’s health-related decision-making autonomy relative to the Comparison Area Findings presented in Papers 7 [7] and 8 [8].	Baseline KPC survey	30-cluster stratified cluster sampling	300 mothers of under-2 children for each survey	Jan 2012 (both Areas A and B)
	Endline KPC survey	30-cluster stratified cluster sampling	300 mothers of under-2 children for each survey	June 2015 (both Areas A and B)
	FGDs	Purposive sampling	Women, men/husbands, mothers-in-law, and Community Health Committees	Jan 2014 (Area A)
	Key informant interviews	Purposive sampling	Community Level-1 Promoters,	May 2015 (both Areas A and B)
	FGDs		*Comunicadoras,* and Self-Help Group participants	
The CBIO+ Approach produces significant increases in community involvement in problem solving relative to a Comparison Area Findings are presented in Paper 8 [8]	Baseline KPC survey	30-cluster stratified cluster sampling	300 mothers of under-2 children for each survey	Jan 2012 (both Areas A and B)
	Endline KPC survey	30-cluster stratified cluster sampling	300 mothers of under-2 children for each survey	June 2015 (both Areas A and B)
	FGDs	Purposive sampling	Women, husbands/partners, mothers-in-law, and Community Health Committee members	Jan 2014
What are the lessons learned in implementing the CBIO+ Approach? How can the CBIO+ Approach be best and most feasibly introduced into the MSPAS framework for health care delivery? Findings presented in Paper 9 [9].	Written questionnaire Key-informant interviews FGDs Group interviews Key-informant Interviews	Purposive sampling	Curamericas/Guatemala staff and MSPAS staff Curamericas/Guatemala staff and MSPAS staff Level-1 Promoters Existing literature about PEC	July 2013 (Area A) Aug 2013 (Area A) June 2015 May 2015 Aug-Nov 2015
How does the cost-effectiveness of the CBIO+ Approach as implemented by Curamericas Global in Guatemala compare to that of other Guatemala maternal and child health programs using different methodologies (based on cost-per-life saved and cost-per- DALY averted)? What are the prospects for scaling up CBIO+ in Guatemala and for implementing and testing it in other countries? Findings presented in Paper 10 [10]	Literature review Cost analysis; LiST (Lives Saved Tool)	Not applicable Not applicable Not applicable	Analysis of Project fiscal records, Project Vital Events Registers, and registers of community census data	Nov 2015 (both Areas A and B)

“Birthing Centers” refers to Community Birthing Centers (*Casas Maternas Rurales*); *FGD* Focus group discussion

### Data quality assurance

Using Excel and Epi Info, all of the data from the baseline and endline KPC surveys for both Areas A and B were cross-checked by Project staff to ensure accurate data entry. The baseline and endline KPC survey findings for both Areas A and B were independently analyzed by two different researchers, providing confidence that the results were accurately reported. Similarly, the anthropometric data arising from these two surveys and also from the September 2012 anthropometric survey were also analyzed independently by two different researchers, again providing confidence that the results were accurately analyzed.

### Contributions of researchers

The Project was most fortunate to have the support of numerous students who assisted in the design of research activities, collection and analysis of data, and writeup of findings. In this article and subsequent one, we list the current affiliations of these researchers. At the time of the data collection these were their affiliations:

- Johns Hopkins University School of Public Health: SM, JL, CW
- Johns Hopkins University School of Arts and Sciences: NM
- University of North Carolina School of Public Health: BL
- Tulane University School of Public Health: KL
- University of Miami School of Medicine: CG
- University of Iowa School of Public Health: EO

## Discussion

We describe here implementation research that is vast in scope in several senses. First of all, there are multiple interventions being tested within a broader approach of a comprehensive programmatic strategy. Secondly, there are multiple sources of data and methods of data collection being employed to carry out the implementation research. Thirdly, by virtue of this paper being a part of a journal supplement that describes the implementation research, there is the opportunity to provide more detail about the context, the strategies used, and the outcomes than would normally be the case.

Implementation research has been defined as “the scientific inquiry into questions concerning implementation – the act of carrying out an intention into effect, which in health research can be policies, programmes, or individual practices (collectively called interventions)” [21]. Implementation research seeks to understand why and how interventions work in the real world and how they might be improved [21]. High-quality implementation research requires a full description of the interventions being implemented, including who is implementing them; the strategies involved, and any deviation from the planned interventions and strategies; the context in which they are being implemented; the outcomes; and the practical and policy implications of the findings [22]. Unfortunately, complete descriptions of implementation research activities are not common. One recent review found only 8% of the peer-reviewed implementation research literature (791 out of 10,292 articles) adequately described the intervention(s) and the set of implementation strategies that accompanied them [22]. Out of these 791 articles, only 28 reported more completely on the above-mentioned descriptions required [22]. Thus, there is a need for implementation research studies to describe the full range of problems, contexts, methods and results more fully in order to reap the full benefits of such research. The studies presented in this supplement can make an important and much-needed contribution to the existing body of implementation research, serving as a comprehensive approach to the monitoring and evaluation of health projects and programs, including those for mothers and children, that can lead to the improvement of their effectiveness.

It would not have been possible to carry out such an extensive implementation research project without the participation of many student volunteers nor without the contributions of Project staff to the data collection process. The series of articles reported here provides the opportunity to describe fully the required features of implementation research described above. This is rarely possible because of space limitations that constrain the reporting of implementation research when they are confined to a single peer-reviewed article.

## Conclusion

The implementation research for the Curamericas/Guatemala Maternal and Child Health Project, 2011–2015, was designed to assess the effectiveness of CBIO+﻿ , which is the expanded set of approaches implemented in conjunction with CBIO, including the Care Group Approach and the Community Birthing Center Approach *–* all integrated with MSPAS services in a rural population of 98,000 Indigenous Maya people in the western highlands of Guatemala. We employed a quasi-experimental mixed-methods design with a comparison area. Data collection methods consisted of multiple household surveys, anthropometric surveys and censuses, vital events registration, verbal autopsies of child and maternal deaths, self-administered questionnaires, FGDs, group interviews, individual interviews, and key-informant interviews. These data collection methods were designed to provide evidence regarding the degree to which the Project improved the population coverage of evidence-based interventions for improving maternal and child health, child nutrition, under-5 and maternal mortality, the quality of clinical care provided at the Birthing Centers*,* the empowerment of women, and the creation of social capital in the community. The findings of these assessment are presented in subsequent papers in this series of articles.

## Data Availability

All of the Project reports, de-identified data, as well as publications about the Expanded CBIO+ Approach cited in this article are available from the corresponding author on request.

## Appendix 1

### Further Details about Classification and Analysis of Causes of Death

The Curamericas/Guatemala Maternal and Child Health Project, 2011–2015, utilized a system of “primary” and “secondary” classifications of cause of death. Primary classifications for child deaths included birth asphyxia, complications of prematurity, acute respiratory infection/pneumonia, diarrhea, sepsis/other infections, and other/miscellaneous causes. Examples of “secondary” causes included “aspiration of meconium” for birth asphyxia or “infant respiratory distress syndrome” for complications of prematurity. “Primary” classifications of the cause of maternal deaths were hemorrhage, pre-eclampsia/eclampsia, sepsis, other direct causes, and indirect causes. A “secondary” classification elucidated the leading attributable cause, such as retained placenta, uterine rupture, or uterine atony for hemorrhage. This system allowed us to harmonize our data with that of the MSPAS.

We also assigned a “delays” classification for both child and maternal deaths, referring to the delays in obtaining appropriate treatment. We modified the Thaddeus and Maine “three-delay” classification scheme [23] for analyzing causes of maternal mortality to a “four-delay” classification scheme for both under-5 and maternal deaths. This involved dividing the Thaddeus and Maine first delay (delay in recognition of a complication) into two parts: (1) delay in recognizing a complication or danger sign, and (2) a delay in deciding to seek treatment at a health facility once a complication or danger sign had been recognized. The last two delays are the same as the last two in the Thaddeus/Maine classification system: (3) delay in transport to a referral facility, and (4) delay in receiving appropriate care once the patient arrived at the facility. The third delay involved transportation – delay in procuring it, or the length of the journey. In the Project Area this was a major issue because of the lack of vehicles and the lack of roads, not to mention the facts that many of the roads that were present were treacherous, all were unpaved, and that the nearest referral hospital was four hours away. The fourth delay involved delay in treatment once arriving at a facility and/or receiving inadequate treatment.

This “four-delay” classification was utilized for two reasons. First, because one of the Project goals was to enable families to recognize and respond to danger signs in children ill with acute respiratory infection/pneumonia or diarrhea as well as to recognize and respond to danger signs during pregnancy, during delivery, or during the postpartum period. Evaluating how many deaths were attributable to the first delay would help assess the penetration of the health education the Project provided through the Care Groups. Also, being able to evaluate “second delays” would help understand the factors that impeded proper care-seeking by the family despite their recognition of the danger. The other reason was that for maternal deaths, MSPAS utilized the same four-delay classification system, and this allowed us to harmonize our maternal mortality data with those of the MSPAS.

Attribution of the delay was carried out by the Institutional Facilitator based on the information gathered during the verbal autopsy. If the family indicated that they recognized the danger but did not respond with prompt care-seeking at a health facility, the Institutional Facilitator inquired why not and recorded the responses. Though the “delays” have traditionally been applied to maternal deaths, the Project elected to apply the delay model to deaths in under-5 children as well to help understand the factors that contributed to these deaths. Therefore, it should be noted that the verbal autopsies provided key qualitative data for the Institutional Facilitator’s analysis of the narrative of the death as conveyed by the family. This helped us to understand the various geographical, socioeconomic, cultural, and gender-based factors that contributed to maternal and child mortality. All questionnaires and forms used for data collection are available from the corresponding author upon request.

## Abbreviations

CBIO: Census-Based, Impact-Oriented
CBIO+: Combination of the CBIO Approach with the Care Group Approach and the Community Birthing Center (*Casa Materna Rural*) Approach
FGD: Focus group discussion
KPC: Knowledge, practice, and coverage (household survey)
M&E: Monitoring and evaluation
MSPAS: Guatemalan Ministry of Public Health and Social Welfare (*Ministerio** de **Salud* *Publica** y **Asistencia** Social*)
PEC: *Programa* *de* *Extensión* *de* *Cobertura*
SIAS: *Sistema Integral de **Atención* *en* *Salud*
